# The Clinicopathological Profile of Eosinophilic Myocarditis

**DOI:** 10.7759/cureus.3677

**Published:** 2018-12-03

**Authors:** Haris Sheikh, Mahrukh Siddiqui, Syed Mohammad Mazhar Uddin, Aatera Haq, Uzair Yaqoob

**Affiliations:** 1 Internal Medicine, Civil Hospital, Karachi, PAK; 2 Surgery, Civil Hospital, Karachi, PAK; 3 Internal Medicine, National Institute of Child Health, Karachi, PAK

**Keywords:** myocarditis, eosinophilic, eosinophilic myocarditis, hypersensitivity, pathology, management

## Abstract

Eosinophilic myocarditis (EM) is a rare form of myocarditis. As there is extreme diversity in its manifestations, the true incidence is difficult to assess and no proper epidemiological criteria are present. It generally presents with a wide array of clinical manifestations. Clinical presentation tends to differ in cases and not all the patients show the same signs and symptoms. The etiology of EM often remains obscure but potential causes have been identified which may include hypersensitivity to drugs, exposure to certain viruses and parasites, and hyper-eosinophilic syndromes. Endomyocardial biopsy is considered to be a gold standard for the diagnosis of EM. Echocardiography, cardiac magnetic resonance, and bio markers particularly serum eosinophilic cationic protein concentrations are also known to aid in diagnosis. EM may lead to progressive, irreversible, and fatal myocardial damage if prompt diagnosis is not made and therapy is not initiated. The current treatment regimens include corticosteroids, cytotoxic agents, and immunosuppressive therapy. However, a proper treatment criterion is yet to be established.

## Introduction and background

Disease name and definition

In 1995, myocarditis was defined by the World Health Organization (WHO)/International Society and Federation of Cardiology (ISFC) as an inflammatory disease of the heart muscle, diagnosed by established histological, immunological, and immunohistochemical criteria [1]. Eosinophilic myocarditis (EM) is a type of myocarditis. It is the cardiac manifestation of hyper-eosinophilia. As the term indicates, it is the inflammation of myocardium due to invasion and subsequent release of toxins by the eosinophils. Hyper-eosinophilic conditions can be classified into three categories based on etiology: (a) idiopathic hyper-eosinophilic syndrome (HES) after exclusion of all known causes; (b) eosinophilia secondary to infections (particularly parasitic or helminthic), medications, allergic disorders, metastatic malignancies, endocrinopathy, and autoimmune disorders; (c) clonal disorders which include acute leukemia, myeloproliferative disorders, and chronic myeloid disorders [2].

Cardiac involvement in cases of peripheral eosinophilia is very common (50%-60%). Eosinophils remain viable in the cardiac tissue for weeks and eventually degranulate releasing toxic substances such as eosinophil-derived neurotoxin, cationic protein, major basic protein, and reactive oxygen species. All these toxins may damage the endothelial cells and myocytes causing necrosis and thrombosis which end up in endomyocardial fibrosis. Inflammatory changes within the endocardium and papillary muscle derangement may affect the heart valves which manifests as regurgitation [2].

## Review

Diagnostic criteria

Cardiac involvement in case of EM may manifest as chest discomfort, dyspnea, marked jugular venous distention, signs and symptoms of mitral or tricuspid regurgitation, S3 or S4 gallop, cool extremities, and peripheral cyanosis [3-5]. These cardiac symptoms are nonspecific and overlap with many other cardiac diseases. However if extra-cardiac symptoms related to eosinophilia also accompany the cardiac manifestations, it might be easy to suspect EM. If myocarditis is suspected, then history should be carefully taken especially past medication history should always be reviewed. Any drug use known to cause eosinophilia should be discontinued instantly. History of travel might be important with emphasis on the regions where eosinophilia-related infections are common. Review whether others in family have eosinophilia suggest a common exposure or familial nature of disease. History of infection or drug use before the onset of symptoms is a very strong indicator of EM and is present in a majority of cases. Drug reaction with eosinophilia and systemic symptoms (DRESS) can occur with sulfasalazine, hydantoin, carbamazepine, D-penicillamine, allopurinol, hydrochlorothiazide and cyclosporine or associated with viral infection (human herpesvirus-6, Epstein-Barr virus, and cytomegalovirus). Patients with DRESS present with fever, rash, systemic involvement, and an appropriate medication history. However, still other patients come with no history of prior infection or allergic diathesis [6].

Due to a wide array of clinical manifestations associated with EM, an appropriate diagnostic criterion has not been established yet. When patients come to the clinic, electrocardiography can be performed after taking a thorough history which may show sinus rhythm, ST segment, and T wave abnormalities. Those who come with signs of infarction may show ST segment elevation, ST segment depression, and Q waves. Presence of Q waves or left bundle branch block is associated with a higher mortality rate [7]. On echocardiography, features of myocarditis include dilated, restrictive, hypertrophic, and ischemic cardiomyopathy. Increased sphericity and left ventricular volume occurs in acute active myocarditis. This procedure is also used to detect the right ventricular involvement, pericardial effusion, and left ventricular thrombus. Right ventricle involvement is a very strong indication of cardiac transplantation [7-8]. Other noninvasive procedures can further aid in diagnosis. These include biomarkers such as troponin T and I, and cardiac magnetic resonance (CMR) imaging. Massively enlarged heart, edema, and pericardial effusion are common findings. However, diagnosis cannot be made entirely on results of CMR imaging because of lack of details of myocarditis, such as the degree of inflammation and the type. Hence, it is logical to perform endomyocardial biopsy (EMB) after CMR [1]. Dallas pathological criteria published in 1986 for the first time presented diagnostic guidelines for the histopathological classification of myocarditis. It proposed that presence of myocyte necrosis along with inflammatory cellular infiltrate on a biopsy specimen is diagnostic of active myocarditis, whereas infiltration not accompanied with myocyte necrosis is borderline myocarditis. Further description about the variant of myocarditis can be made based on the quality of the cellular infiltrate, whether it is eosinophilic, lymphocytic, or granulomatous [1, 7].

Epidemiology

Eosinophilic myocarditis has a predilection for males. This is due to the fact that female sex hormones play an important role in providing protection against the infectivity of myocytes and at the same time also decrease potentially harmful myocardial inflammatory responses. In contrast male hormones have an inhibitory effect on the anti-inflammatory responses leaving males susceptible to such diseases [9-10]. Mean age for all forms of myocarditis is reported to range from 20 to 51 years. However, children can also be a victim of this disease [9]. Myocarditis has been found to be the cause of sudden deaths in up to 12% of cases in autopsy studies of young adults and athletes [9]. As there is extreme diversity in its manifestations, the true incidence of EM is difficult to assess and no proper epidemiological criteria have been established yet.

Clinical manifestations

Eosinophilic myocarditis can present both as an acute or a chronic condition. Patients with acute EM experience a rapidly declining cardiac course due to its acute nature. Acute EM has a poor prognosis culminating in death within weeks of illness onset. In contrast, patients with the more commonly occurring chronic EM usually present with congestive heart failure which responds very well to glucocorticoid therapy and cytotoxic agents. Chronic myocarditis usually occurs in the setting of a systemic disease leading to eosinophilia [11].

Eosinophilic myocarditis generally presents with a wide array of clinical manifestations. One of the most common complaints which bring the patient to the clinic is chest discomfort and pain [12]. A positive history of allergies and use of certain drugs is also found in a majority of patients [13]. Moreover, a past history of infection a few weeks or months before the abrupt onset of chest pain and fatigue has also been observed [11,13]. Cardiovascular manifestation includes signs and symptoms of congestive heart failure (congestive cardiomyopathy), for example low cardiac output, cool extremities, peripheral cyanosis, low pulse volume, raised jugular venous pressure, mitral regurgitation, tricuspid regurgitation, and a massively enlarged heart. Moreover, conduction delays, ST and T segment abnormalities, and S3 or S4 gallop have also been reported [3-5]. Myocardial fibrosis after active inflammation can lead to arrhythmias [5]. Cardiomegaly, pulmonary congestion, pleural effusion, and pericardial effusion all have been seen on chest X-rays [13].

Acute necrotizing EM is an aggressive form of EM with an acute onset, a short course, and a high mortality rate [7, 11]. Initially, it appears to be a mild viral infection with myocardial invasion dominated by mononuclear cells. Myocardial necrosis and eosinophilic infiltration is common, and hence, the name. However, the pathophysiology of EM related to this viral origin remains obscure and more research on this etiology needs to be done [14]. Eosinophilia is present but with massive migration of these eosinophils into the tissues, there is a decrease in the peripheral eosinophil count [15]. Thus, there are no prominent extra-cardiac symptoms [11]. Myocyte death caused by necrosis leads to heart failure. Myocardial fibrosis may occur which further puts the patients at risk for fatal arrhythmias [13]. Presenting complaints range from dizziness, episodes of syncope (due to sudden hemodynamic compromise), severe dyspnea (due to acute congestive heart failure), shock, and sometimes infarction like pattern on electrocardiography [11, 15]. Acute necrotizing EM is known to have three stages: an acute necrotic stage, a thrombotic stage, and a fibrotic stage. Acute coronary syndrome can occur in any of the three phases [12]. Cerebral thromboembolic events are common during the second stage. Unfortunately, anticoagulative agents may not be entirely beneficial [12].

Characteristic features of hypersensitivity myocarditis which are either drug induced or secondary to infection; include rash, fever, sinus tachycardia, and eosinophilia. This variant of myocarditis is also associated with eosinophilic and lymphocytic infiltration of myocardium but myocyte necrosis is very uncommon. Therefore, this entity does not have a debilitating disease course. Withdrawal of the medication improves the condition in most cases [16]. Patients with hypersensitivity myocarditis are not critically ill at the time of death and cause of death is mostly arrhythmias rather than myocardial failure. Far-stretched myocardial necrosis is not seen but there is indication of vasculitis in small myocardial vessels. Most conspicuous extra-cardiac pathology is inflammatory eosinophilic hepatitis [12, 15]. Hypersensitivity myocarditis is a self-limiting condition. It is not associated with ST-segment abnormalities. Cardiac involvement is rare with only mild heart failure symptoms that resolve spontaneously [12].

Idiopathic HES is characterized by peripheral eosinophilia of >1500 μL over six months without a known cause [12, 17]. Eosinophilic myocarditis occurs in more than 50% of the people with idiopathic HES. Cardiac involvement is the main cause of morbidity and mortality in most cases [13]. Unlike hypersensitivity myocarditis, myocyte necrosis is common in idiopathic HES followed by extensive endocardial scarring and restrictive cardiomyopathy [13]. Idiopathic HES has overlapping cardiac manifestations with all the other known causes of EM such as hypersensitivity myocarditis, Churg Strauss, malignancy, etc. But again there are certain differences, for example cardiac involvement if present is only mild in hypersensitivity myocarditis, whereas it might be severe enough to cause death in a patient with idiopathic HES [17].

In an early review of 65 cases of idiopathic HES, the most common presenting symptom was dyspnea, occurring in 60% of the patients. Some 75% of the 55 patients who could be evaluated had signs and symptoms of congestive heart failure and 4% had evidence of pericarditis. In the same review of 26 HES patients followed prospectively, 42% had dyspnea, 27% had chest pain, 12% had cough, 8% had palpitations, and 4% had embolic events . These patients were subsequently found to have mitral regurgitation (42%), congestive heart failure (38%), aortic regurgitation (4%), and aortic stenosis (4%). Although myocardial infarction is a rare complication of HES, it may occur preceded by an embolic event due to endomyocardial fibrosis and thrombus formation in the left ventricular outflow tract [17]. Major basic protein contained in the eosinophils is a potent stimulator of platelets and hence an increased risk of thrombi formation in EM [15]. Hyper-eosinophilic syndromes whether idiopathic or secondary to a known cause, may involve multiple organs along with the heart. Cutaneous manifestations include maculopapular or pustular eruption, angioedema, and pruritis. Gastrointestinal abnormalities such as diarrhea and malabsorption may also be found [3-4]. Hepatosplenomegaly is seen in a number of cases with tenderness. Nervous system involvement which signifies a poor prognosis may present as transient ischemic attack or cerebrovascular accident, diffuse central nervous system dysfunction, and peripheral neuropathy [3].

Etiology and pathogenesis

The etiology of EM is not always distinct, but in several cases, it has been identified. It is found to be associated with hypersensitivity, parasitic infections, HESs, asthmatic bronchitis, and mycoplasma pneumonia [18].

Eosinophilic myocarditis is a rare clinical commodity, and it is usually discovered at postmortem examinations. Numerous studies revealed that EM was seen in 0.5% of unselected autopsy series and in more than 20% of explanted hearts from heart transplant recipients [19]. This condition is characterized pathologically by diffuse or focal myocardial inflammation with eosinophilic infiltrates, and mostly occurs in association with peripheral blood eosinophilia [20].

Eosinophilic myocarditis can present as an acute or chronic condition. Acute necrotizing myocarditis is the initial presentation of disease; it is rare and often fatal, followed by endocardial thrombosis, and chronic endomyocardial fibrosis [21]. Chronic myocarditis ranges mostly from one to 11 years from diagnosis till death. These people often develop symptoms of congestive heart failure [22].

EM possesses three stages. In stage 1, there is acute necrotic stage due to the infiltration and extra cellular deposition of eosinophil, and consequently interleukin 5 mediated injury. This stage lasts for about two to three weeks. Stage 2 is the thrombotic stage, characterized by layered thrombus along damaged endocardium due to an activation of tissue factor by eosinophils. Cerebral thromboemboli are common during this thrombotic stage. Stage 3 is considered as the fibrotic stage which is characterized by myocardial fibrosis [12].

Myocardial infiltrates vary in their distribution, as they could be either perivascular or interstitial. Furthermore, both epicardium and myocardium can also be involved. This can result in various features like myocyte necrosis, fibrosis, granuloma formation, and fibroid necrosis of collagen [13]. Numerous studies have stated about eosinophilic granule proteins leading to progression of endomyocardial lesions. This is due to the fact that eosinophilic granules contain a variety of substances including a collagenase that cleaves type 1 and type 3 collagen, major basic protein, eosinophilic cationic protein, acid phosphatase, aryl sulfatase, ribonuclease, peroxidase, B-glucuronidase, and alkaline phosphatases [23-24]. This can lead to myocyte necrosis, and can result in increased permeability and inhibition of mitochondrial respiration [13]. Below are the descriptions of some of the well-defined etiological factors.

Hypersensitivity Eosinophilic Myocarditis

Hypersensitivity myocarditis is a rare disease [25], which is known to be a cardiac manifestation of delayed type hypersensitivity, mostly caused by drug exposure. However, the incidence is elevated in heart transplant candidates [26]. Furthermore, the patients suffering from this disease as a result of drug exposure are characterized by an interstitial inflammatory infiltrate, perivascular infiltrate, vasculitis, very little necrosis, absence of fibrosis, and hepatic involvement [13]. Also, these patients are known to die from arrhythmias [14]. Numerous drugs from various classes are involved including antibiotics (ampicillin), anti-inflammatory (indomethacin), diuretics (acetazolamide), and others (dobutamine) (Table 1) [5, 13].

**Table 1:** Drugs capable of causing hypersensitivity myocarditis. Modified from an article written in 2006 [13].

Antibiotics
Amphotericin B Ampicillin Chloramphenicol Cephalosporin Penicillin Streptomycin Tetracycline
Anti-inflammatory
Indomethacin Oxyphenbutazone Phenylbutazone
Anti-convulsants
Antituberculous Carbamazepine Para-aminosalicylic acid Phenindione Phenytoin
Diuretics
Acetazolamide Chlorthalidone Hydrochlorothiazide Spironolactone
Sulfonamides
Sulfadiazine Sulfisoxazole
Others
Amitryptyline Captopril Digoxin Dobutamine Enalapril Methyldopa Sulfonylurea Tetanus toxoid

A more severe form of hypersensitivity is termed as acute eosinophilic necrotizing myocarditis. It is characterized by an extreme eosinophilic infiltration, marked edema, myocyte necrosis, and it often follows a more fulminant course. Such patients develop a severe heart failure within few days [13, 27].

Viral Eosinophilic Myocarditis

Several viruses are known to cause myocarditis in humans. However, Coxsackie virus B3, being an enterovirus is the most dominant etiological agent in human beings. Both the innate and adaptive immune response determines the severity of myocardial damage. Furthermore, both the immune responses contribute to the development of chronic myocarditis and dilated cardiomyopathy. The hallmark of the disease is the presence of a mononuclear infiltrate [12, 28-30].

Parasitic Eosinophilic Myocarditis

Some parasites can also become an etiological agent for myocarditis. One example is visceral larva migrans (VLM) which is caused by the ingestion of *Toxocara canis* (*T. canis*), the most common round worm of dogs and cats. Myocarditis can occur in about 10%-15% cases of VLM [31]. Once eggs of *T. canis* reach the human gastrointestinal tract, they hatch and enter the portal system thereby reaching the liver. Some larvae can migrate to the lungs and heart through the systemic circulation [32]. As the larva grows, it punctures through the vessels and can initiate an immune response. Myocarditis in VLM can occur from direct larval invasion to the myocardium and/or hypersensitivity reactions [33]. The characteristics of parasitic myocarditis include granulomatous reaction with increased eosinophils. Furthermore, myocyte destruction can occur due to migrating larvae, inflammation, and repair [34].

Eosinophilic Myocarditis Associated with Bronchial Asthma

The diagnosis of asthma or chronic obstructive pulmonary disorders is often made easily, and with good treatment available, most of the patients lead a comfortable life. However, certain complications can occur. One such rare complication is EM. In such a case, eosinophilic infiltrate accumulates in both the heart and lungs. Furthermore, EM can also occur in asthmatic patients who have started their treatment with anti-leukotriene drugs after withdrawal of steroids [18-19].

Eosinophilic Myocarditis Associated with Hyper-eosinophilic Syndromes

Hyper-eosinophilic syndromes involve eosinophilic infiltration of the myocardium, and it comes under idiopathic HES. Cardiac disease occurs in more than 50% of patients with idiopathic HES (defined as an absolute eosinophil count greater than 1.5 × 109/L lasting for more than six months in the absence of any known cause of hyper-eosinophilia and involves heart, nervous system, and/or bone marrow). The HES includes many diseases like Davies’ endomyocardial fibrosis and Loffler’s myocarditis. Cardiac diseases are the major cause of morbidity and mortality in patients having this syndrome [13, 35].

Eosinophilic Myocarditis Associated with Vaccine Administration

Numerous studies indicate that EM can also occur after administration of certain vaccines including meningococcal C and hepatitis B in children [36].

Diagnostic considerations

Clinical presentation of EM is mostly nonspecific and has a wide spectrum. In majority of the cases, the diagnosis is done by a combination of criteria such as a preceding infection of the upper respiratory tract, electrocardiography (ECG), echocardiography, CMR studies, biomarkers, imaging, and EMB [5, 13].

Electrocardiography

Electrocardiography can be used to monitor ST and T abnormalities, sinus tachycardia, and arrhythmias which occur mostly in all forms of EM [5, 13].

Echocardiography

Echocardiography allows the various estimates of heart function like cardiac output, ejection fraction, wall thickness, chamber size, and systolic and diastolic function. It is one of the most valuable tools in order to rule out other cardiomyopathies [5, 13]. Before an EMB, echocardiography is done to exclude pericardial effusion and intra-cavitatory thrombi [9]. Patients with fulminant myocarditis mostly have normal cardiac chamber sizes with an increased septal thickness, whereas patients with acute myocarditis often have marked left ventricular dilation with normal wall thickness [37].

Cardiovascular Magnetic Resonance

The CMR study is the only noninvasive method to view the extent of endomyocardial involvement for diagnosis and treatment of EM. It is highly specific and sensitive than EMB. As CMR fails to give information about the degree of inflammation and the type of myocarditis (for e.g., eosinophilic, giant cell), complete diagnosis of myocarditis cannot be done. As a result it seems appropriate to perform biopsy following CMR in patients suspected with myocarditis [1, 38].

Biomarkers

Numerous biomarkers can also confirm the diagnosis of EM. Eosinophilic granules consisting of eosinophilic cationic proteins (ECP) along with other variety of substances lead to endomyocardial lesion in the area of cardiac injury [23-24]. The serum ECP concentration can be used as a valuable indicator for diagnosis and treatment of EM [39]. Furthermore, troponin I and T levels are elevated in majority of the cases of acute EM. Troponin I is more specific than Troponin T, but Troponin T is more sensitive [7].

Imaging

X-Ray and computed tomography (CT) scan can be used to monitor chest findings which usually show cardiomegaly, pulmonary edema, and pleural effusion. These are important in both asthmatic and nonasthmatic EM [5, 13].

Endomyocardial Biopsy

Endomyocardial biopsy is often considered as a gold standard for the diagnosis of EM [18-19]. It gives the description about the variant of myocarditis based on the quality of the cellular infiltrate, whether it is eosinophilic, lymphocytic, or granulomatous. It should never be delayed if the results of the imaging techniques are not diagnostic with clinical suspension [40]. However despite being a valuable tool, EMB can have certain drawbacks. Sometimes it is not a very sensitive technique as the infiltrates in EM are often focal [41]. Furthermore as EMB is too invasive, it cannot be performed in all patients [39]. Due to lack of facilities and clinical experience, EMB is infrequently used for the diagnosis of myocarditis. However, when this procedure is performed by experienced individuals, it is much safer with a complication rate of below 1 % [1].

Management

Eosinophilic myocarditis is rarely diagnosed and is usually first discovered at autopsy [19]. The beneficial and clinically effective treatment of EM is linked to proper understanding of the etiology and underlying pathogenesis of the disease. Data addressing the issue of management are scarce and focus on a few important points. The management of EM includes withdrawal of the offending agent and starting standard treatment for heart failure. Cardiac support and treating the underlying disease is generally considered.

Corticosteroid Therapy

Corticosteroid therapy in the early stage can have a favorable effect if early diagnosis is made. When an EMB identifies early stage EM, the disease can be treated with corticosteroids which might prevent cardiac damage [31, 42]. The CMR studies of EM patients treated with steroids have revealed marked reduction of subendocardial late gadolinium enhancement representing acute inflammation and necrosis. Corticosteroid therapy in the early stage may prevent further progression to the intermediate thrombotic-necrotic stage with mural thrombi and the fibrotic stage which has been postulated as the final stage in the time course of EM [35]. Some patients with EM have documented dramatic responses to steroid therapy, including those with a history of severe left ventricular dysfunction [43].

Patients with chronic EM usually present with symptoms of congestive heart failure. These patients respond well to glucocorticoids. In contrast, acute necrotizing eosinophilic cardiomyopathy follows a more rapidly deteriorating cardiac course with less systemic involvement. Glucocorticoids have not been found to be effective, primarily because they have been introduced late in the clinical course [12].

Treatment with Mepolizumab

Mepolizumab is a humanized blocking monoclonal antibody against IL-5. Recent studies have shown that mepolizumab has significant effects at stabilizing blood eosinophil counts. These effects are clinically relevant, given that reducing eosinophil levels is currently the primary treatment goal for patients with the HES and its associated myocarditis and that long-term corticosteroid therapy is associated with a range of undesirable side effects [44].

Treatment with Anti-helminthic Agents

Acute myocarditis occasionally progresses to a fulminant course. Most of these fulminant cases are caused by viral infections for which corticosteroids are unyielding [42]. VLM, described by Beaver et al. in 1952 results mainly from infection by the common roundworms of dogs and cats [45]. Infection with the parasite usually causes marked eosinophilia and the development of eosinophilic-rich granulomatous lesions in the soft tissues of the body, including the myocardium [34].

Albendazole is a benzimidazole anti-helminthic agent. It is used as a treatment for various parasitic infections. The mechanism of its anti-helminthic action is inhibition of tubulin polymerization and microtubule-dependent glucose uptake inhibition. Prompt anti-helminthic treatment for parasitic infection is recommended in EM associated with VLM, so albendazole should be administered as early as possible in the clinical course of patients presenting with VLM [32].

Treatment with Cytotoxic Agents

Randomized control trials are yet to be performed to evaluate the proper and effective treatment of EM. Although observational trials have reported that cytotoxic agents may be beneficial in the efficacious and sufficient treatment of EM [12].

As described by Loffler, the sub-acute and chronic forms of EM characterized by variable degree of fibrosis and thrombosis and chronically progressive heart failure are more well known. Cytotoxic agents are known to improve outcome in these patients [20].

Immunosuppressive Therapy

The role of immunosuppressive therapy in the treatment of EM remains slightly controversial. Immunosuppressive therapy has been known to prevent recurrence of EM [43]. Aggarwal et al. reported a successful use of a combination of steroids and azathioprine at a dose of 2 mg/kg in a patient diagnosed to have EM who presented with cardiogenic shock [46].

Recent studies have shown that an early, appropriate inflammatory response has a beneficial effect in patients with myocarditis. However, in some patients, heightened T-cell activity may be injurious and detrimental. Thus, appropriately timed immunosuppressive treatment would be helpful in some patients with myocarditis, if it prevented or mitigated the harmful T-cell response [47].

Cardiac Care

Pharmacological cardiac care should include anticoagulant and antiplatelet drugs which have been advocated in the treatment of acute necrotic eosinophilic endomyocardial disease [48]. Beta blockers, angiotensin-converting enzyme (ACE) inhibitors, and α-blockers such as carvedilol or metoprolol are also used to prevent cardiac damage. Diuretics are sometimes needed to maintain optimum ventricular filling pressures [11]. Finally, for the treatment of fulminant EM, mechanical ventricular support is often used along with ionotropic agents to regulate and maintain proper myocardial contractility [27].

Prevention Against Drugs Inducing Hypersensitivity

There are numerous drugs and drug classes that have been associated in causing the hypersensitivity form of EM. Treatment should include withdrawal of all these etiologically relevant drugs and agents (Table 1).

Ventricular Assist Device

The risk of ventricular tachycardia and heart block is high in inflammatory cardiac diseases. Refractory ventricular tachycardia and ventricular fibrillation may require placement of a ventricular assist device (VAD) for temporary circulatory support. Alternative treatment options for ventricular fibrillation may include extracorporeal membrane oxygenation or a total artificial heart. VADs have been used as a successful bridge to recovery in giant-cell myocarditis and lymphocytic myocarditis. However, their use in the potential recovery in necrotizing EM is novel [11].

Surgery

In selected cases, cardiac surgery (endocardectomy) and transplants have been performed [20].

Prognosis

Eosinophilic myocarditis is an extremely rare disease with poor prognosis, and it is potentially fatal. Due to its progressive course and diagnostic difficulties, most of the patients are diagnosed upon autopsy without prompt treatment before their death [9, 19].

1. In acute necrotizing EM, patients develop severe heart failure within days to a week. A pericardial effusion is seen in 75% of cases, occasionally causing tamponade. The mortality rate exceeds 50% and the median survival is only a few days [27]. The patient is usually healthy, experiences a rapid and acute progression to systolic failure, and hemodynamic compromise [11].

2. Release of major basic protein in acute necrotizing EM is also a potent stimulator of platelets that may result in cardiac thrombi [15].

3. Cerebral thromboemboli are very common during the thrombotic stage of EM. Unfortunately, anticoagulative therapy may not be entirely protective [20].

4. Myocyte death caused by necrosis and apoptosis in EM is responsible for the development of heart failure. Furthermore, myocardial fibrosis can occur despite treatment and puts the patient at a high risk for future fatal arrhythmias [13].

5. Endocardial fibrosis has become a hallmark of EM as the disease progresses. Diastolic dysfunction is always seen and restrictive cardiomyopathy is evident in the chronic stage. EM in the chronic stage is characterized by the obliteration of the apex in right ventricle or left ventricle by thrombosis and fibrosis [35].

Early, quick, and correct diagnosis is, therefore, absolutely essential to treat the disease and prevent fatality.

## Conclusions

Doctors, clinicians, and healthcare professionals should consider uncommon disorders such as EM in patients presenting with acute cardiomyopathy symptoms and ventricular tachycardia when the clinical course is progressive, despite standard cardiac care. There is a lack of proper and definitive diagnostic criteria and management guidelines. Although the disease is rare it is potentially fatal and extensive studies are needed to improve prognosis and provide better health care and relief to the affected individuals. Most of the data on EM are in the form of either autopsy reports or sporadic case reports. Establishing a definite epidemiological criteria and annotating genetic basis if any, is needed. Furthermore, there is an unmistakable need for advanced methods of diagnosis, and clinical trials to establish proper management criterion.
